# An Immune Signature Robustly Predicts Clinical Deterioration for Hepatitis C Virus-Related Early-Stage Cirrhosis Patients

**DOI:** 10.3389/fmed.2021.716869

**Published:** 2021-07-19

**Authors:** Cheng Guo, Chenglai Dong, Junjie Zhang, Rui Wang, Zhe Wang, Jie Zhou, Wei Wang, Bing Ji, Boyu Ma, Yanli Ge, Zhirong Wang

**Affiliations:** ^1^Department of Gastroenterology, Tongji Hospital, School of Medicine, Tongji University, Shanghai, China; ^2^Department of Thoracic and Cardiovascular Surgery, Tongji Hospital, School of Medicine, Tongji University, Shanghai, China

**Keywords:** hepatitis C virus, cirrhosis, immune microenvironment, prognosis, prediction

## Abstract

Hepatitis C virus (HCV)-related cirrhosis leads to a heavy global burden of disease. Clinical risk stratification in HCV-related compensated cirrhosis remains a major challenge. Here, we aim to develop a signature comprised of immune-related genes to identify patients at high risk of progression and systematically analyze immune infiltration in HCV-related early-stage cirrhosis patients. Bioinformatics analysis was applied to identify immune-related genes and construct a prognostic signature in microarray data set. Gene Ontology (GO) and Kyoto Encyclopedia of Genes and Genomes (KEGG) functional enrichment analyses were conducted with the “clusterProfiler” R package. Besides, the single sample gene set enrichment analysis (ssGSEA) was used to quantify immune-related risk term abundance. The nomogram and calibrate were set up via the integration of the risk score and clinicopathological characteristics to assess the effectiveness of the prognostic signature. Finally, three genes were identified and were adopted to build an immune-related prognostic signature for HCV-related cirrhosis patients. The signature was proved to be an independent risk element for HCV-related cirrhosis patients. In addition, according to the time-dependent receiver operating characteristic (ROC) curves, nomogram, and calibration plot, the prognostic model could precisely forecast the survival rate at the first, fifth, and tenth year. Notably, functional enrichment analyses indicated that cytokine activity, chemokine activity, leukocyte migration and chemotaxis, chemokine signaling pathway and viral protein interaction with cytokine and cytokine receptor were involved in HCV-related cirrhosis progression. Moreover, ssGSEA analyses revealed fierce immune-inflammatory response mechanisms in HCV progress. Generally, our work developed a robust prognostic signature that can accurately predict the overall survival, Child-Pugh class progression, hepatic decompensation, and hepatocellular carcinoma (HCC) for HCV-related early-stage cirrhosis patients. Functional enrichment and further immune infiltration analyses systematically elucidated potential immune response mechanisms.

## Introduction

For approximately 1.6% (range: 1.3–2.1%) population with positivity for anti-hepatitis C virus (HCV) antibodies worldwide (1). Removing either spontaneously or as a result of antiviral treatment, the global viraemic prevalence (positive for HCV RNA) is estimated at 1% (range: 0.8–1.14%) individuals with HCV infection in reality (1). Meanwhile, 50–80% of HCV infection patients develop into chronic hepatitis C (1). Chronic HCV infection is the primary cause of liver cirrhosis, especially in the developing world (2). Liver cirrhosis affects hundreds of millions of people worldwide, causing more than one million deaths in 2010 (3). Cirrhosis is the major driver in the development of hepatocellular carcinoma (HCC). Liver cirrhosis develops from ongoing fibrosis injury and eventually leads to liver failure and HCC. HCC incidence in HCV-related cirrhosis was extremely high (up to 7% per year) (2). Mechanically, fibrosis results from the breakdown of the dynamic balance between extracellular matrix deposition and degradation in chronic diseases of the liver and other parenchymal organs (4). Until recent years, treatment for HCV with direct-acting antivirals (DAAs) regimens were associated with moderate success but were challenging to tolerate (5).

In fact, it was uncertain that at what stage cirrhosis becomes irreversible, but irreversibility becomes more likely as extracellular matrix collagen deposition (6). Generally, the prognosis of fibrosis mainly depends on early detection and clinical intervention. It was clear that early diagnosis and timely intervention can prevent or reverse the decompensation process (7). Importantly, patients with early cirrhosis, which is more common than liver cancer, lack a valid clinical prognostic marker.

Thus, we developed a robust immune-related prognostic index for patients with HCV-related early-stage cirrhosis who never developed HCC or cirrhosis complications at the enrolled time. In addition, our work showed the immune microenvironment and immune functionalities.

## Materials and Methods

### Data Collection

Gene expression profiling of liver needle biopsy specimens and clinical information from 216 patients with hepatitis C–related Child–Pugh class A cirrhosis were available at National Center for Biotechnology Information Gene Expression Omnibus (GEO; GSE15654; https://www.ncbi.nlm.nih.gov/geo/query/acc.cgi?acc=GSE15654). External validation, GSE54100 (https://www.ncbi.nlm.nih.gov/geo/query/acc.cgi?acc=GSE54100) was performed using archived liver biopsy specimens from 145 patients with HCV-related compensated cirrhosis who had a liver biopsy and were followed at Massachusetts General Hospital. Another validation cohort GSE54099 (https://www.ncbi.nlm.nih.gov/geo/query/acc.cgi?acc=GSE54099) was performed using formalin-fixed, paraffin-embedded (FFPE) tissues sections (10 micron-thick) sliced from FFPE blocks from 90 HCV-related patients. All HCV infection was confirmed by serum HCV antibody and/or RNA. External independent validation dataset GSE54100 (*n* = 145) and GSE54099 (*n* = 90) with common clinicopathological characteristics. Besides, transcriptome profiling and the related clinical materials of HCC patients (*n* = 371) were obtained from The Cancer Genome Atlas (TCGA, https://cancergenome.nih.gov/) database.

### Identification of Immune-Related Genes Related to the Prognosis of HCV-Related Early-Stage Cirrhosis Patients

Immune-related genes were obtained using the Immunology Database and Analysis Portal (ImmPort) database (https://immport.niaid.nih.gov) (8). Then, the immune-related genes expression matrix was extracted via matching immune genes in the primary GEO expression matrix. Subsequent analysis in the relationship between immune-related genes expression matrix and 216 HCV-related early-stage cirrhosis patients survival information was conducted *via* univariate Cox regression analysis.

### Construction and Validation of an Immune-Related Prognostic Signature

Immune-related prognosis genes screened by univariate Cox regression were then analyzed using the least absolute shrinkage and selection operator (LASSO) regression analyses method (9). Then four genes were screened out. Further, a prognostic signature was built by performing multivariate Cox regression analysis using the selected genes. Previous researches (10) were employed for the determination of the risk score for every patient applying the formula below: Risk score = coef_gene1_^*^ expr_gene1_ + coef_gene2_^*^ expr_gene2_ + … + coef_genen_^*^ expr_genen_. A linear integration of the expression levels of genes weighted by regression coefficients (coef) was used to assign the risk score. Log transformation of the hazard ratio (HR) from the multivariate Cox regression analysis was employed to calculate the coef value. Patients enrolled in the training group were fallen into the groups of high-risk and low-risk based on the median score as a cutoff value. The Kaplan–Meier (K-M) method and log-rank tests with the “survival” R package were utilized to compare the survival rate between high-risk and low-risk groups based on the median risk score. Besides, the signature's effectiveness was assessed by performing the area under the curve (AUC) value of the time-dependent receiver operating characteristic (ROC) curves using “survivalROC” packages (11).

### The Relationship Between the Prognostic Signature and Other Clinical Outcomes

Information of clinical deterioration terms including hepatic decompensation, progression of Child–Pugh class and HCC were extracted from GEO dataset. Patients were divided into high and low group based on the median risk score. The cumulative incidence of these clinical endings was calculated and drawn using GraphPad Prism (v. 8.0.1). *P*-value and Hazard ratio were also performed.

### Independence of the Prognostic Signature From Traditional Clinical Characteristics

Univariate and multivariate Cox regression analyses were explored to determine whether the immune-related prognostic signature was an independent factor compared with clinical characteristics (presence of varices, bilirubin ≥ 1.0 mg/dl and platelet < 100,000/mm^3^) in HCV early-stage cirrhosis patients.

### Construction and Validation of a Predictive Nomogram

The construction of nomogram from clinical factors was made from multivariate regression analysis in the 216-patient cohort. The assessment of the discrimination and calibration of the predictive nomogram was made by applying the concordance index (C-index) and the calibration curve. The construction of nomogram and calibrate was made by the “rms” package (12). Besides, the signature's accuracy was assessed by performing ROC curve using “survivalROC” package (11).

### GO and KEGG Pathways Enrichment Analyses

To explore the mechanisms whereby the identified immune-related prognostic signature may influence HCV-infected early-stage fibrosis patient clinical outcomes, Gene Ontology (GO) and Kyoto Encyclopedia of Genes and Genomes (KEGG) functional enrichment analyses were conducted with the “clusterProfiler” R package (13). GO functional enrichment analysis including biological process (BP), cellular component (CC), and molecular function (MF). The top 10 GO terms and the top 20 KEGG pathways were identified as being significant using the “ggplot2” R package (14). The statistical significance threshold of functional enrichment analysis was set at an adjusted *p*-value < 0.05.

### ssGSEA Analysis

To explore immune cell infiltration, immune pathway activity, and functionality in HCV-related compensated cirrhosis patients, the single sample gene set enrichment analysis (ssGSEA) was conducted to built immune-related term enrichment scores. The ssGSEA function in the “gsva” R package was used to quantify immune cell infiltration based on the expression level of immune cell-specific marker genes (15). Scores corresponding to 29 different immune-related terms, including innate and adaptive immune cells, were determined for HCV-related early-stage cirrhosis patients. Type I and type II interferon response family genes, plasmacytoid dendritic cell precursors (pDCs) family genes, immune cell markers, and checkpoint genes expression levels were assessed in different clusters. Subsequent visualize differences analysis in the distributions of immune terms in the low- and high-risk patient groups via the “vioplot” R package (16).

### Statistical Analysis

All statistical analyses were conducted using Rstudio (v.1.4.1106) and GraphPad Prism (v. 8.0.1). Continuous data are given as medians or as means ± standard deviation (SD). *P* < 0.05 was considered statistically significant.

## Results

Identification and comprehensive analysis of immune genes related to prognosis in HCV-related early-stage cirrhosis patients.

To develop a prognostic index, we download genomic data and clinical information from the GEO website. Clinical variates of the enrolled HCV-related early-stage cirrhosis patients were summarized in Table 1. Two hundred sixteen patients were enrolled, and 52 patients have esophageal/gastric varices, 108 patients with bilirubin ≥ 1.0 mg/dl, and 99 patients with platelet < 100,000/mm^3^. After a median follow-up time of 10 years, 66 patients died, 71 patients developed decompensation, 66 patients developed Child-Pugh B or C, and 65 patients developed to HCC. Genomic expression profiling was extracted from GSE15654. A total of 1,250 immune-related genes were matched in the microarray data. Then, we performed univariate Cox regression to explore the relationship between the expression profiles of the 1,250 genes and 216 patients with survival information. The results indicated that 156 of 1,250 genes were significantly associated with the prognosis of HCV-related early-stage cirrhosis patients (*p* < 0.05, Supplementary Table 1).

**Table 1:** Clinical features of 216 hepatitis C–related early-stage cirrhosis patients.

**Characteristic at enrollment**	***n* (%)**
Presence of varices^a^	
YES	52 (26)
NO	159 (74)
Bilirubin ≥ 1.0 mg/dl	
YES	108 (50)
NO	108 (50)
Platelet <100,000/mm^3^	216
YES	99 (46)
NO	117 (54)
**Clinical outcome**	
Death	66 (31)
Hepatic decompensation^b^	71 (34)
Progression of Child–Pugh class	66 (31)
HCC	65 (30)

a *The varices information could not be calculated in 4 patients due to missing data*.

b *The hepatic decompensation information could not be calculated in five patients due to missing data*.

**Figure 1:**
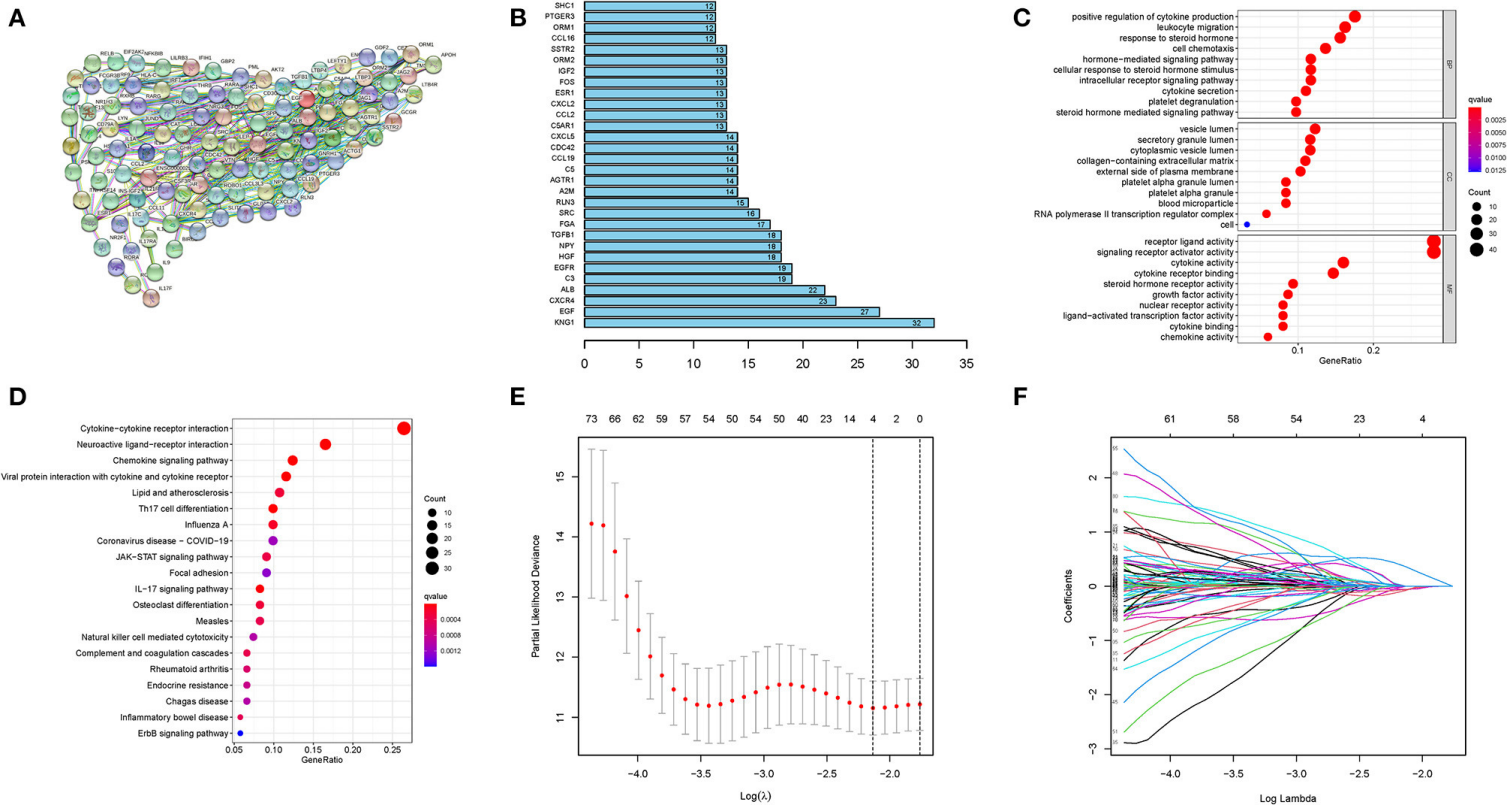
Construction of prognostic model. **(A)** PPI network and **(B)** the top 30 genes with enriched functional partners. **(C)** GO and **(D)** KEGG bubble graph. **(E)** and **(F)** LASSO Cox regression.

Protein-protein interaction networks (PPI) of the selected 156 genes were analyzed using the website tool STRING (https://string-db.org) (Figure 1A). The top 30 genes with enriched functional partners were performed (Figure 1B). Next, GO, and KEGG functional enrich analyses were conducted with the “clusterProfiler” R package. Enriched biological processes (BP), including cell chemotaxis, leukocyte migration, leukocyte chemotaxis, T cell activation, platelet degranulation, positive regulation of cytokine production, and cytokine secretion (Figure 1C). Meanwhile, cytokine activity, cytokine receptor binding, growth factor activity, chemokine activity, and cytokine binding were the regular molecular function (MF) (Figure 1C). KEGG analysis showed that cytokine–cytokine receptor interaction, chemokine signaling pathway, IL-17 signaling pathway, and viral protein interaction with cytokine and cytokine receptors were common pathways in the collected genes (Figure 1D).

These results indicated vital roles of immune-related terms, including cell chemotaxis, cytokine activity, and inflammation signaling pathway, in the progress of HCV-related cirrhosis.

### Establishment and Validation of a Prognostic Signature

Based on the LASSO regression with 10-fold cross-validation, we screened four genes (CXCL2, ENG, GHR, and ORM2) from 156 selected genes with a repetition frequency >900 times in 1,000 substitution samplings (Figures 1E,F). Further multivariate Cox regression was applied and finally determined three genes (ENG, GHR, and ORM2) of the four genes to construct a prognostic index (Table 2). The prognostic risk score = (1.291 ^*^ expression level of ENG) + (−0.568 ^*^ expression level of GHR) + (−0.597 ^*^ expression level of ORM2). In the signature, the positive coefficient of ENG suggesting that it may be a risk factor because its high expression is related to poor prognosis. However, the high expression of GHR and ORM2 may be protective factors, considering their expression levels were related to longer survival time.

**Table 2:** Three-gene identified using Cox regression analysis and the LASSO regression method.

**Id**	**Coefficient**	**Hazard ratio**	***P*-value**
ENG	1.291	3.635	2.304e−06
GHR	−0.568	0.567	0.021
ORM2	−0.597	0.550	0.024

**Figure 2:**
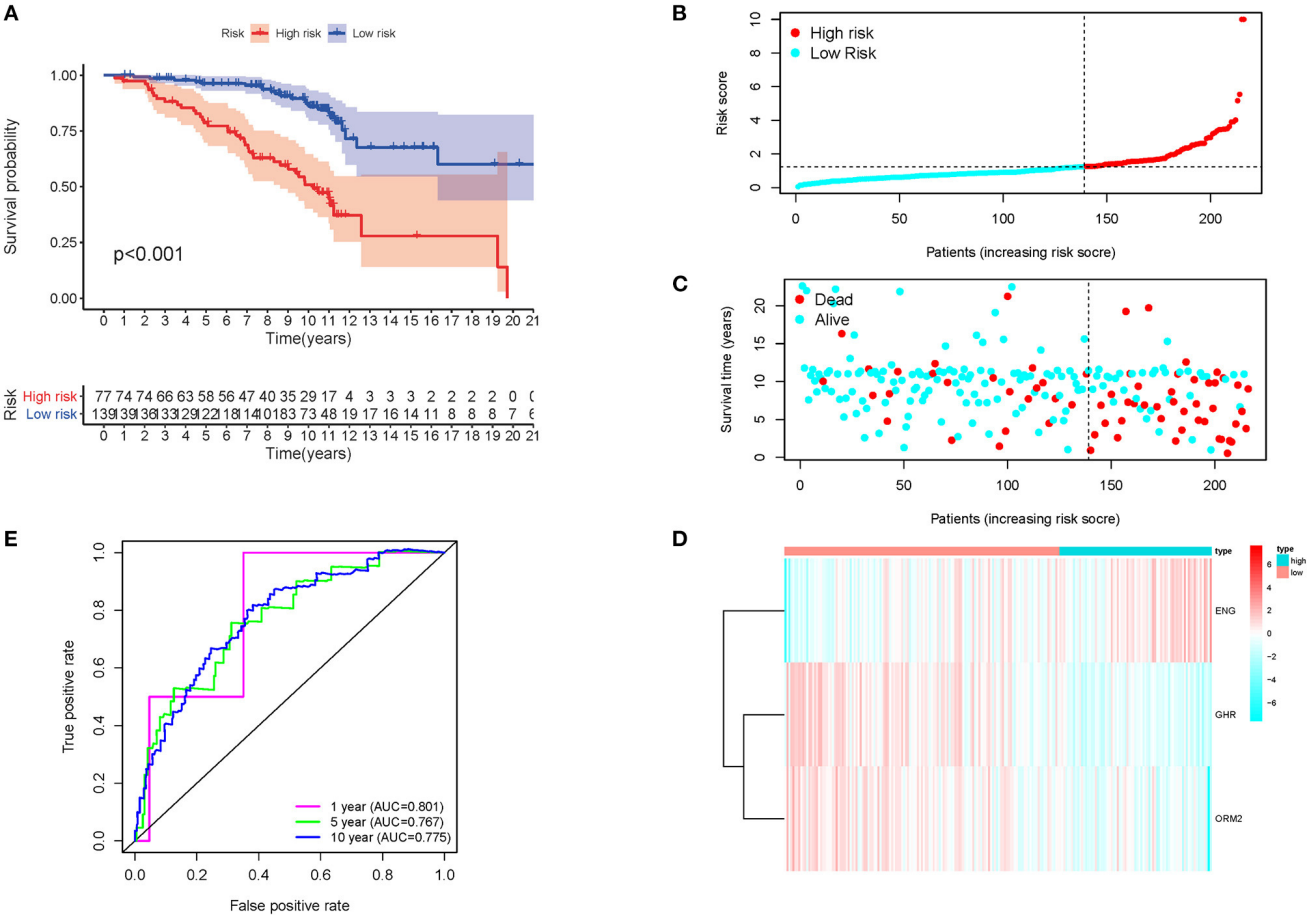
K-M survival analysis, risk score distribution, and time-dependent ROC curves of a prognostic model in the HCV-related early-stage cirrhosis cohort. **(A)** K-M survival curve analysis between high and low-risk groups. The distribution of **(B)** immune risk score, **(C)** survival status, and **(D)** expression matrix of the three genes. **(E)** The time-dependent ROC curve analysis for measuring the predictive performance on OS.

The above formula was adopted to obtain the risk score of every member in the database, and the median-risk score was used to cluster these patients into different groups as the cutoff value. The group with a higher score was called the high-risk group, and the other group was called the low-risk group. According to the K-M analysis, overall survival (OS) was significantly worse in the high-risk group than in the low-risk group. (*p* < 0.001) (Figure 2A). The distribution of immune risk score, survival status, and expression matrix of the three genes for patients with HCV-related early-stage cirrhosis was performed in Figures 2B–D. Besides, AUC in the time-dependent ROC curve analysis reached 0.801, 0.767, and 0.775 at 1, 5, and 10 years, respectively (Figure 2E), indicating robust specificity and sensitivity of the prognostic signature in predicting survival.

### The Relationship Between the Prognostic Signature and Other Clinical Deterioration

**Figure 3:**
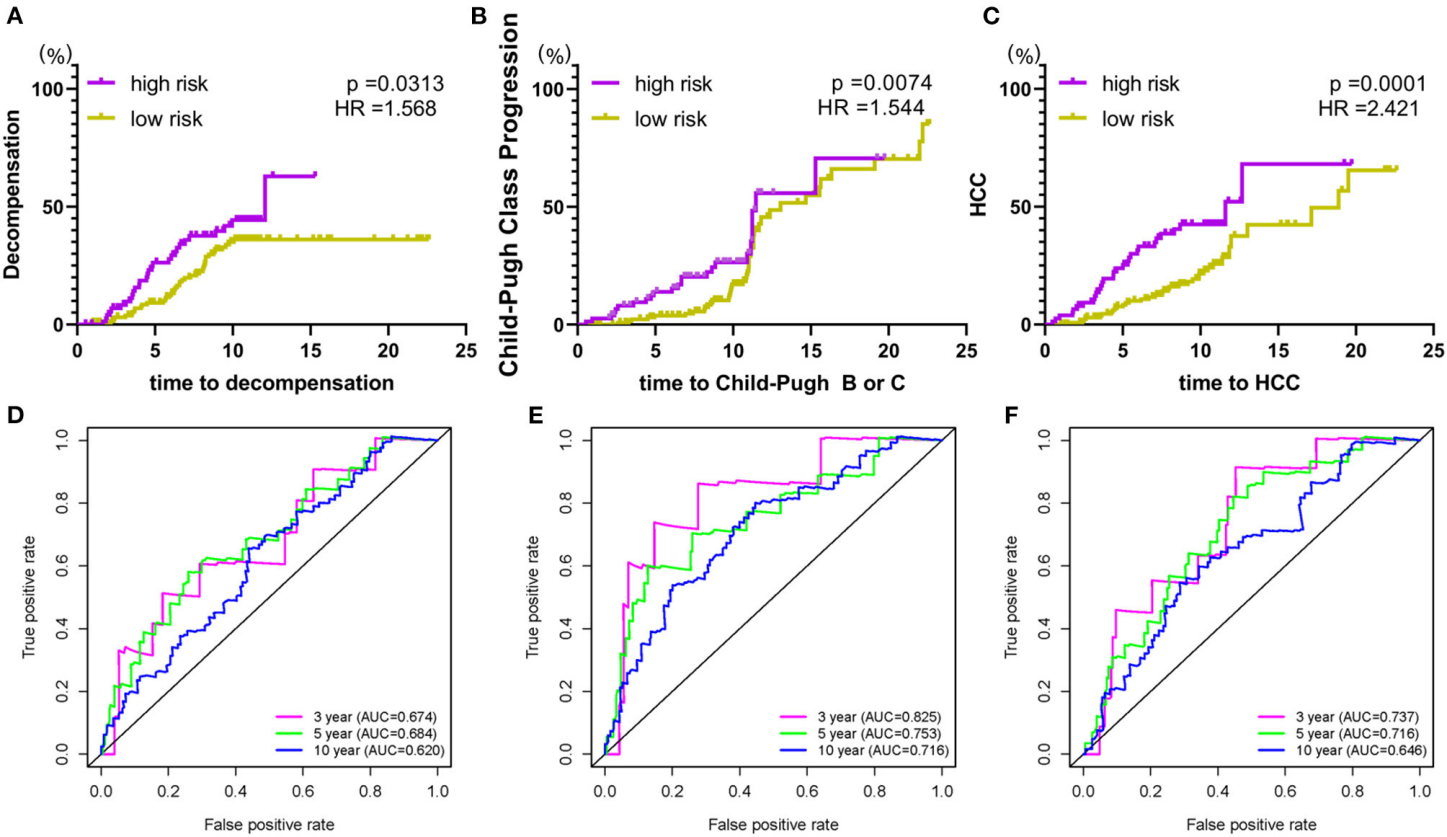
The prognostic signature is associated with poor clinical endings. **(A)** risk score and hepatic decompensation; **(B)** risk score and Child-Pugh class progression; **(C)** risk score and HCC. **(D–F)** The time-dependent ROC curve analysis for verification of the index on different malignant clinical events. Hazard ratios and p-values were given for the difference between the high and low-risk groups.

To further expand the application spectrum of the signature, the cumulative incidence of the clinical outcomes, including hepatic decompensation, progression of Child-Pugh class, and HCC were calculated. Surprisingly, a robust separation between the high and low-risk groups was performed. Patients in the high-risk group have a high incidence of poor clinical endings (hepatic decompensation, *p* = 0.0313, HR = 1.568; progression of Child-Pugh class, *p* = 0.0074, HR = 1.544; HCC, *p* = 0.0001, HR = 2.421; Figures 3A–C). In addition, time-dependent ROC curves were performed to validate sensitivity and specificity of the index in predicting malignant clinical events at 3, 5, 10 years, respectively (Figures 3D–F). These results revealed that the prognostic signature could be used in different clinical outcomes prediction with high efficiency, which benefits clinical applications. The reliable accuracy of the prognostic biomarker could help screen out early-stage cirrhosis patients who benefit from preventive interventions to alleviate cirrhosis complications.

### The Relationship Between Risk Score and Clinical Parameters

**Figure 4:**
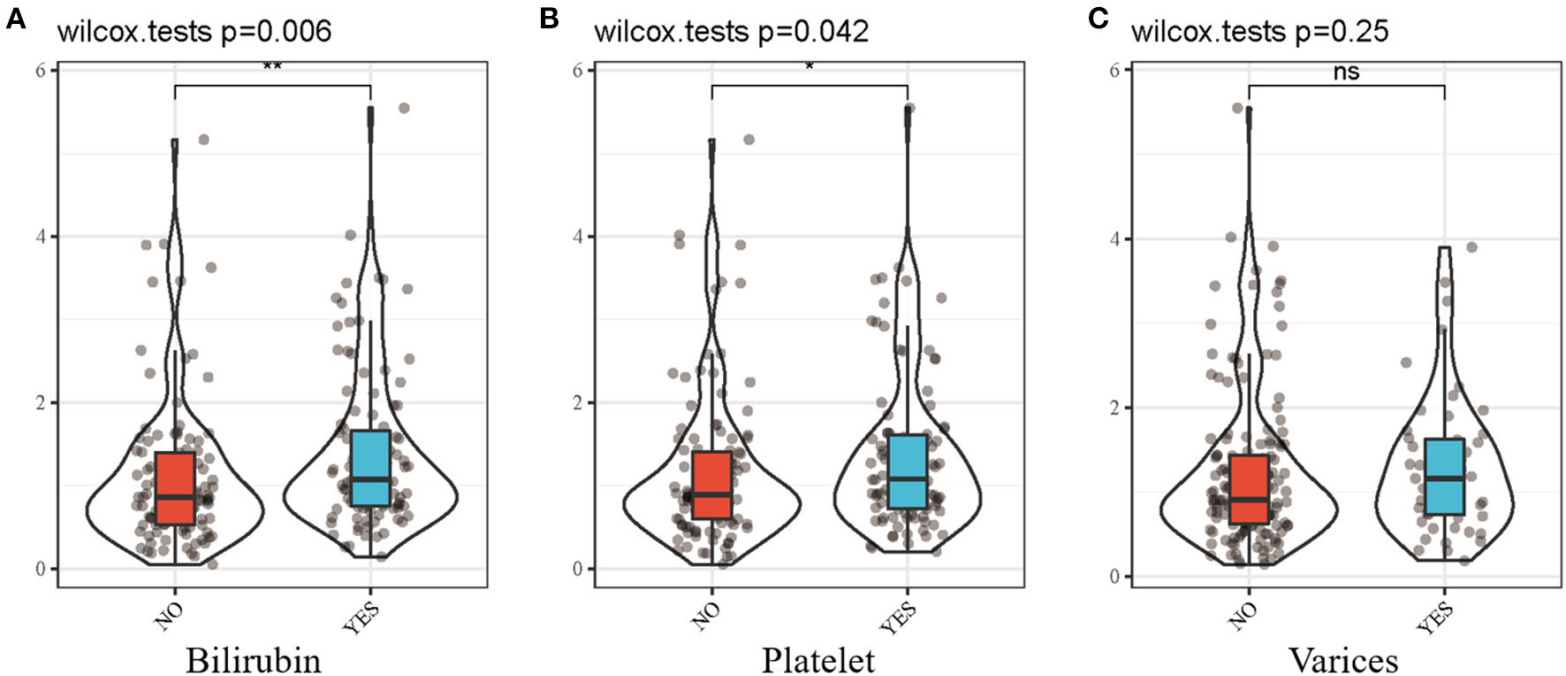
Differential analysis between risk score and clinicopathological features. **(A)** risk score and bilirubin. **(B)** risk score and platelet. **(C)** risk score and varices. Bilirubin, bilirubin ≥ 1.0mg/dl; Platelet, platelet < 100,000/mm^3^; Varices, presence of varices. *p < 0.05; **p < 0.01.

Moreover, we investigated the differences in the prognostic signature scores in different subgroups of clinical parameters. The results revealed that risk score was significantly different in bilirubin ≥ 1.0 mg/dl (*p* = 0.006; Figure 4A) and platelet < 100,000/mm^3^(*p* = 0.042; Figure 4B). There was no significant difference in the presence of varices between the two groups (*p* = 0.25; Figure 4C). A higher gene score was found in more serious clinical parameters and advanced disease stages.

### Association of Prognostic Signature and Immune Infiltration

**Figure 5:**
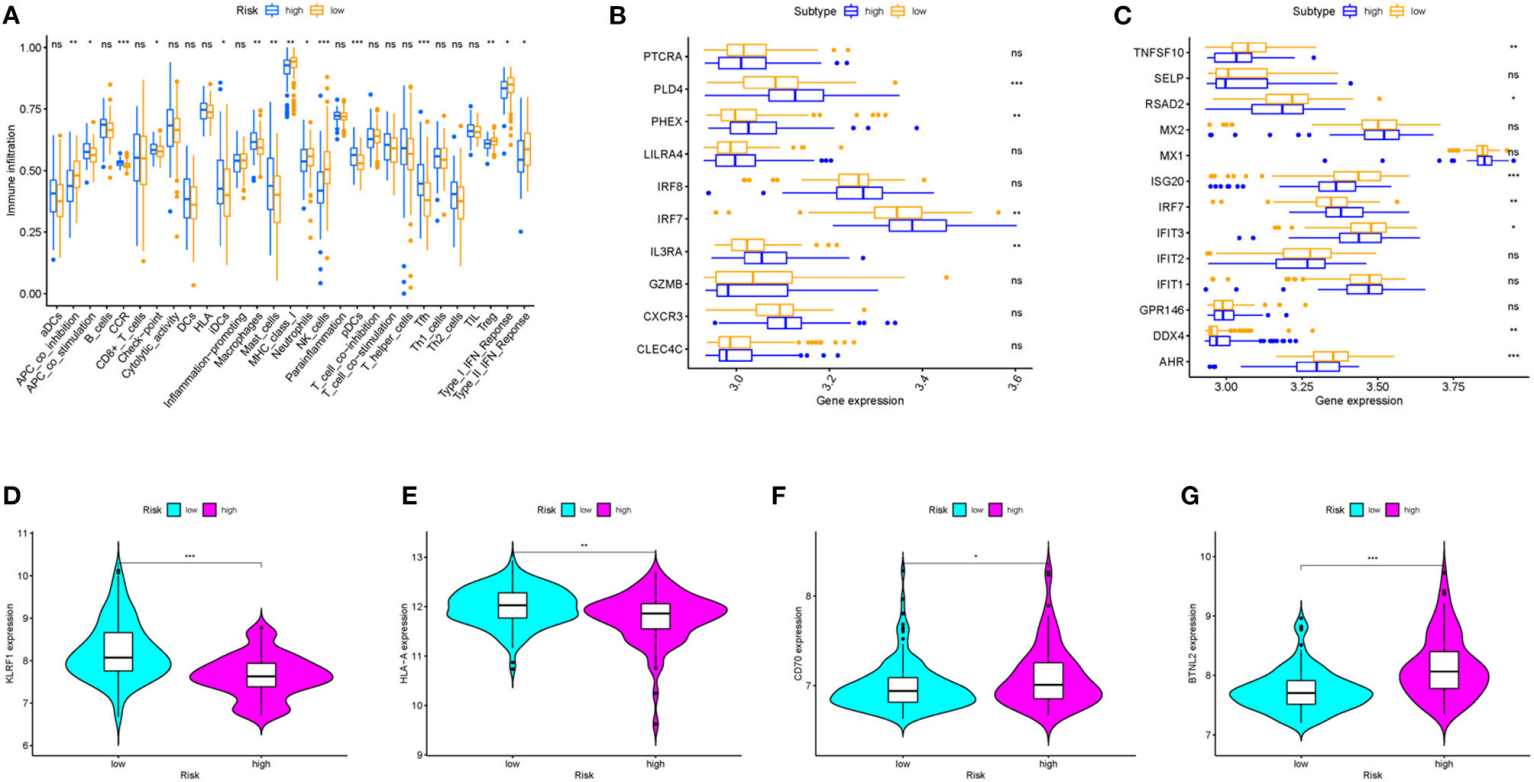
The relationship between prognostic signature, immune cell infiltration, and immune functionality. **(A)** The relative enrichment of 29 immune-related risk terms in high- and low-risk HCV-related early-stage cirrhosis patients. **(B)** pDCs family genes and **(C)** type I and type II IFN response family genes expression profiles in high and low-risk groups. **(D–G)** KLRF1, HLA-A, CD70, and BTNL2 expression levels in different groups.

Immune cell types, immune functions, or pathways were enrolled to assess immune cell infiltration among HCV-related early-stage cirrhosis patients in an integrated fashion via ssGSEA analysis of transcriptome profiling. Immune cell infiltration plays a vital role in HCV infection. Twenty-nine immune items were incorporated into this analysis, and 15 of these immune items differed significantly between the high- and low-risk groups in the overall patient cohort (Figure 5A). Among these significantly different items, antigen-presenting cell (APC) co-stimulation, CCR, checkpoint, immature dendritic cells (iDCs), macrophages, mast cells, pDCs, and follicular T helper cells (Tfh) were positively related to the risk score. However, the expression of MHC class I, neutrophils, natural killer (NK) cells, Treg, APC co-inhibition, type I interferon (IFN) response, and type II IFN response was opposite.

Then, we explored the further relationship of the expression of pDCs family genes and type I and type II IFN response family genes and risk score (Figures 5B,C). All differently expressed type I and type II IFN response family genes in patients with low-risk scores were higher expressed than patients with high-risk scores except for IRF7 and DDX4. IFN has important antiviral activity and immunomodulatory function in HCV infection and autoimmunity disease (17, 18). IFN upregulates the antiviral and immune regulatory activities of IFN-stimulated genes (ISGs) through binding to its receptor (19, 20). Moreover, patients in the high-risk group were related with a significantly lower expression level of KLRF1, one marker of NK cells (Figure 5D). NK cells play an important role in alleviating liver fibrosis through activation of metabotropic glutamate receptor 5 or Siglec-7 expression (21–23). Patients with end-stage cirrhosis often lack NK cells and have a weak response to cytokine stimulation (24). In addition, the expression of HLA-A, one marker of MHC class I cells, and CD70 and BTNL2, members of check-points genes, were also performed (Figures 5E–G). These findings served systematic analysis of immune infiltration in HCV-related early-stage cirrhosis.

### Nomogram Construction and Validation

To determine whether the predictive prognostic signature was independent of other clinical characteristics, we performed univariate and multivariate Cox regression analyses. Univariate and follow-up multivariate analyses showed that bilirubin ≥ 1.0 mg/dl (*p* < 0.001), platelet < 100,000/mm3 (*p* < 0.05) and risk score (*p* < 0.001) were independent elements for the poor prognosis of the HCV-related early-stage cirrhosis cohort (Table 3).

**Table 3:** Univariate and multivariate Cox regression analyses.

	**Univariate analysis**			**Multivariate analysis**		
	**HR**	**95% CI**	***P***	**HR**	**95% CI**	***P***
Varices	1.946	1.138–3.325	0.015	1.646	0.944–2.872	0.079
Bilirubin	3.295	1.908–5.690	<0.001	2.98	1.667–5.327	<0.001
Platelet	3.168	1.799–5.581	<0.001	1.985	1.095–3.598	0.024
Risk score	1.167	1.097–1.242	<0.001	1.192	1.111–1.278	<0.001

**Figure 6:**
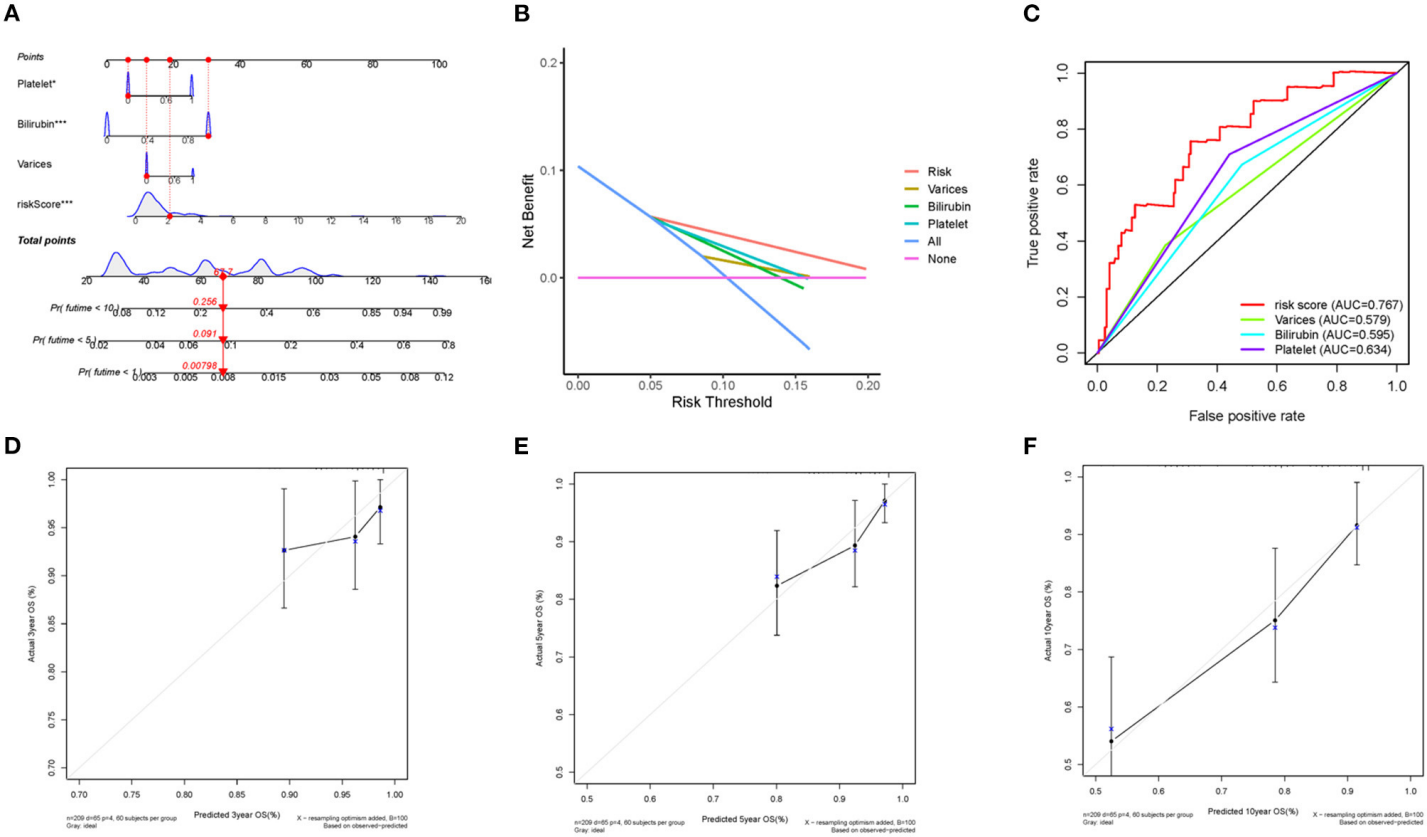
Nomogram construction and validation. **(A)** the predictive nomogram. **(B)** the decision curve analysis and **(C)** ROC curve for the signature and clinical parameters. **(D)** 3-year, **(E)** 5-year and **(F)** 10-year calibration curves for OS.

Next, a nomogram was built by combining clinical various of varices, bilirubin, platelet and risk score (Figure 6A). Each parameter in the nomogram was assigned a specific score. Based on the actual situation of every sample, the score related to every prognostic element to get the total score, which corresponded to the corresponding scale. The survival rates of patients at the first, fifth, and tenth year could be obtained. By measuring the extent of fit between the C-index forecast by the nomogram in the standard curve and the baseline time, the predictive ability of the nomogram model could be evaluated and quantified. The C-index was 0.737 (95% CI:0.671–0.802) for the nomogram, with 1,000 cycles of bootstrapping. Clinical usefulness of the prognostic model was estimated by decision curve analysis (DCA), which was a plot of the “Net Benefit” against “Risk Threshold Probabilities.” The higher Net Benefit value, the more patients benefit. It was clear that, compared to clinical parameters, including varices, bilirubin, and platelet, the risk model has a better Net Benefit in a wide risk threshold at 5 years (Figure 6B). Moreover, according to the time-dependent ROC curve analysis, the AUC value for the immune-related prognostic signature was 0.767 at 5 years, which was higher than the AUC values for presence of varices (AUC = 0.579), bilirubin ≥ 1.0 mg/dl (AUC = 0.595), and platelet < 100,000/mm3 (AUC = 0.634) (Figure 6C). In order to estimate calibration of the nomogram model, we performed calibration curves at the third, fifth and tenth year. Although the performance of the calibration curves at the third year was poor, the performance at the fifth year was better than that at the third year, and with the extension of follow-up time, the calibration curves of the nomogram showed great consistency between the predicted OS rates and actual observations at the tenth year (Figures 6D–F). These results indicated that the nomogram model did not perform well in short-time prediction, but show robustly ability in long-time survival prediction. Our work focuses on the prognosis of compensated cirrhosis, which usually has a longer survival time than decompensated cirrhosis and liver cancer.

**Figure 7:**
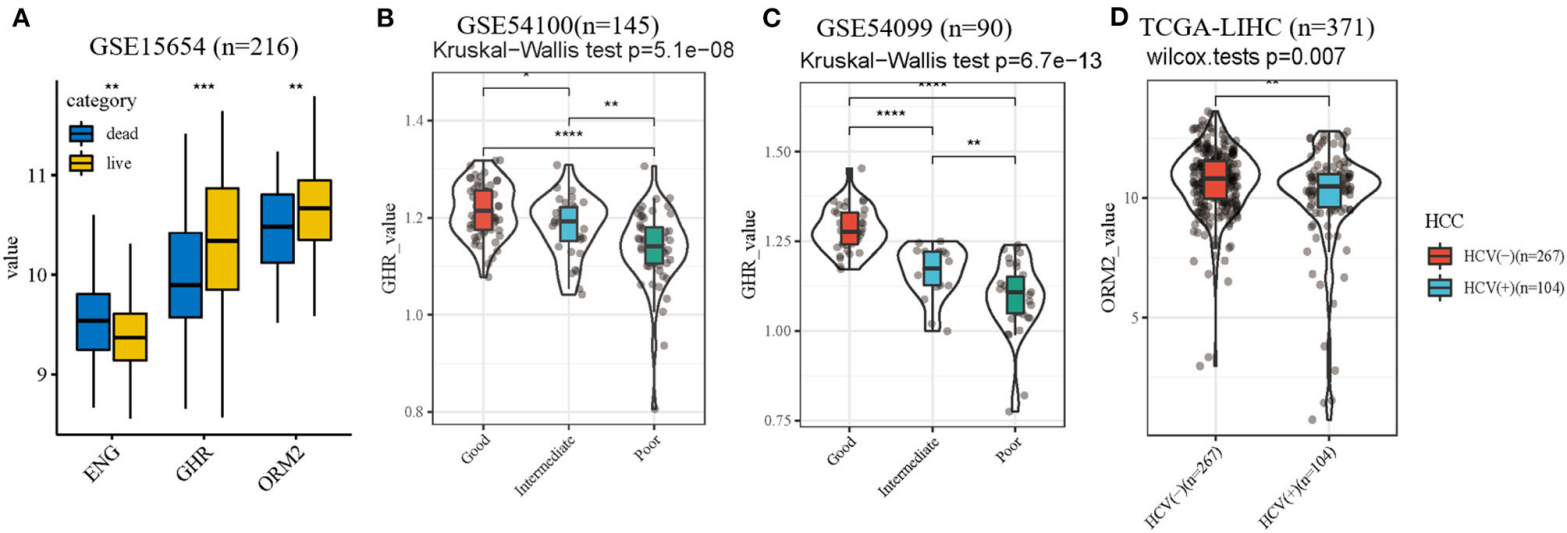
Internal and external datasets verification. **(A)** expression levels of the three genes in patients with different survival status. **(B,C)** the association of GHR value with different clinical outcomes in external independent datasets. **(D)** different ORM2 value of HCC patients with HCV infection or not. *p < 0.05; **p < 0.01; ***p < 0.001; ****p < 0.0001.

Besides, distinct expression levels of the three genes in patients with different survival status in training cohort were performed (Figure 7A). To further validate the accuracy of the prognostic index, external independent datasets were enrolled. The relationship between the expression level of GHR gene and prognostic stratification in GSE54100 (*n* = 145) and GSE54099 (*n* = 90) were performed (Figures 7B,C). These results verified GHR was a protective factor in HCV-related cirrhosis development. In addition, due to limited data resources, we analyzed the differential expression of ORM2 in HCC patients with HCV infection or not (Figure 7D). HCC patients with HCV infection have a lower expression level of ORM2 compared with non-HCV infection HCC patients.

## Discussion

In the absence of highly sensitive and accurate molecular prognostic biomarkers for HCV-related compensated cirrhosis patients, we developed a satisfactory 3-gene signature, which can successfully predict the patients' clinical outcomes. More importantly, this signature can also accurately predict Child-Pugh class progression, hepatic decomposition, and development to HCC, which means the signature was a sensitive measure of the severity of HCV-related cirrhosis and lethality even in compensated patients. Our work revealed the ability of the index in predicting the tendency of liver cirrhosis to worsen. This model revealed a close association between HCC and liver failure state by Child-Pugh class classification progression compared with other HCC prognostic markers (25).

Besides, based on the immune-related prognostic index, our finding further revealed that patients with different risk score stratifications have distinctly different immune microenvironments, which may explain different clinical endings. In the pathogenesis of cirrhosis, the immune system not only plays the role of immune-mediated inflammatory mechanisms, persistent inflammatory stimulation and cell damage promote fibrogenesis through activate hepatic stellate cells and cause immune dysfunction (26). Innate immune cells, including Kupffer cells, mast cells, and resident masters, are the first defense mechanisms against pathogens. However, with the deterioration of cirrhosis, the antibacterial function of circulating neutrophils and monocytes is gradually damaged, end-stage cirrhosis patients were susceptible to bacterial infection (27). Meanwhile, chronic viral infection led to persistent specific immune inflammation stimulation, which aggravating fibrosis and may cause acute compensatory dysfunction and liver failure, both of which are associated with high short-term mortality. Systemic inflammation (elevated steady-state immune cell activation and circulating inflammatory mediators) aggravates hemodynamic derangement and kidney injuries, regularly occurring in patients with acute-on-chronic liver failure (28). An initial systemic inflammatory response (cytokine storm) works as a trigger of the systemic inflammation leading to a compensatory anti-inflammatory response that impairs resistance to infection. Cirrhosis patients with immunodeficiency accompany systemic inflammation were regarded as cirrhosis associated immune dysfunction (CAID) (26). In fact, the mechanism of damage and pathogen-associated molecular patterns activating immune cells and promoting systemic inflammatory response, with ongoing fibrosis progression, is complex, which has not been fully understood.

Direct-acting antivirals (DAAs) has made rapid advance in chronic HCV infections therapy (5, 29). IFN-based therapies, which are no longer recommended, have been replaced by DAA regimens through different viral elimination mechanisms (30). Sustained virological response (SVR) was defined as undetectable serum HCV RNA at least 12 weeks posttreatment (31). SVR after DAA treatment was regarded as closely associated with long-term clinical benefits (31). Thus, almost all stage patients with chronic HCV infection should be treated with DAAs, including patients with decompensated cirrhosis (32–35). Emerging large cohorts further support the results.

DAAs treatment inhibits IFN-λ production and recover exogenous IFN-α reactivity. Besides, it was reported that DAAs downregulated interferon-stimulated genes (ISGs) induced by Hepatitis C virus infection and alleviate HCV-induced extrahepatic symptoms (36).

In addition, there were still some limitations in the research. First, the clinical application of molecular biomarkers based on gene expression has been challenging because of the poor reproducibility of measurements (37). Second, the prognostic model was established relying on the GEO dataset only. Further laboratory experiments and clinical trials are needed to validate the reliability of the results.

## Conclusion

Conclusively, through a comprehensive analysis of the GEO data set, our work identified an immune-related genes index with accurate and efficient clinical deterioration prediction for HCV-related early-stage cirrhosis patients. Besides, enriched immune function and pathways of prognostic immune genes were exhibited. In addition, we elucidated systematic analysis of the immune microenvironment in cirrhosis patients in different risk groups. However, informatics analysis alone is not sufficient to verify the validity of the indicators and to research possible molecular immune mechanisms of HCV-related compensated cirrhosis. Thus, subsequent lab experiments and follow-up experiments will devote to uncover the mechanism involved in HCV-related cirrhosis.

## Data Availability Statement

Publicly available datasets were analyzed in this study. This data can be found at: https://www.ncbi.nlm.nih.gov/geo/query/acc.cgi?acc=GSE15654, https://www.ncbi.nlm.nih.gov/geo/query/acc.cgi?acc=GSE54100, https://www.ncbi.nlm.nih.gov/geo/query/acc.cgi?acc=GSE54099, https://cancergenome.nih.gov/, and https://immport.niaid.nih.gov.

## Ethics Statement

The gene expression profiling and corresponding clinical information in this study were obtained from GEO and TCGA databases and were freely available to the public, which means this study does not require ethical approval from an Ethics Committee.

## Author Contributions

CG, YG, and ZW designed the study. CG, CD, JZ, RW, JZ, ZW, BJ, and BM complicated and analyzed data. CG and CD wrote this manuscript. All authors have read and approved the final manuscript.

## Conflict of Interest

The authors declare that the research was conducted in the absence of any commercial or financial relationships that could be construed as a potential conflict of interest.

## Abbreviations

HCV: hepatitis C virus
HCC: hepatocellular carcinoma
DAAs: direct-acting antivirals
GEO: gene expression omnibus
ImmPort: the immunology database and analysis portal
LASSO: the least absolute shrinkage and selection operator
GO: gene ontology
KEGG: Kyoto encyclopedia of genes and genomes
BP: biological process
CC: cellular component
MF: molecular function
ssGSEA: the single sample gene set enrichment analysis
PPI: protein-protein interaction networks
SVR: sustained virological response
ISGs: interferon-stimulated genes.
