# Supplementary material for: An Immune Signature Robustly Predicts Clinical Deterioration for Hepatitis C Virus-Related Early-Stage Cirrhosis Patients
Source: Front Med (Lausanne). 2021 Jul 19;8:716869. doi: 10.3389/fmed.2021.716869 (PMC8326446; doi:10.3389/fmed.2021.716869)
Supplement: Supplementary file 1 [file Table_1.DOCX]

**Supplementary Table S1. 156 immune-related genes significantly associated with the prognosis of HCV-related early-stage cirrhosis patients**

| gene | HR | HR.95L | HR.95H | p-value |
| --- | --- | --- | --- | --- |
| A2M | 0.554 | 0.341 | 0.898 | 0.017 |
| ACO1 | 0.693 | 0.494 | 0.971 | 0.033 |
| ACTG1 | 3.451 | 1.351 | 8.817 | 0.010 |
| AGTR1 | 0.634 | 0.403 | 0.997 | 0.049 |
| AKT2 | 0.669 | 0.469 | 0.953 | 0.026 |
| ALB | 0.420 | 0.267 | 0.662 | 0.000 |
| AMELX | 1.359 | 1.017 | 1.817 | 0.038 |
| APOH | 0.415 | 0.256 | 0.674 | 0.000 |
| AQP9 | 0.610 | 0.403 | 0.924 | 0.020 |
| AR | 0.357 | 0.185 | 0.687 | 0.002 |
| AZGP1 | 0.454 | 0.235 | 0.878 | 0.019 |
| BIRC5 | 3.611 | 1.186 | 10.988 | 0.024 |
| BMP1 | 0.578 | 0.374 | 0.892 | 0.013 |
| C3 | 0.357 | 0.182 | 0.700 | 0.003 |
| C5 | 0.367 | 0.211 | 0.640 | 0.000 |
| C5AR1 | 1.806 | 1.176 | 2.774 | 0.007 |
| CAT | 0.446 | 0.251 | 0.792 | 0.006 |
| CCL11 | 1.391 | 1.028 | 1.882 | 0.033 |
| CCL16 | 0.370 | 0.210 | 0.653 | 0.001 |
| CCL18 | 0.392 | 0.164 | 0.937 | 0.035 |
| CCL19 | 1.911 | 1.086 | 3.361 | 0.025 |
| CCL2 | 1.617 | 1.011 | 2.585 | 0.045 |
| CCL3L1 | 2.170 | 1.065 | 4.422 | 0.033 |
| CD1E | 2.544 | 1.353 | 4.784 | 0.004 |
| CD244 | 1.447 | 1.043 | 2.008 | 0.027 |
| CD3G | 1.572 | 1.039 | 2.379 | 0.032 |
| CD79A | 1.689 | 1.019 | 2.800 | 0.042 |
| CDC42 | 1.359 | 1.053 | 1.755 | 0.018 |
| CETP | 0.528 | 0.364 | 0.764 | 0.001 |
| CGB2 | 13.432 | 4.241 | 42.545 | 0.000 |
| CGB5 | 1.359 | 1.095 | 1.685 | 0.005 |
| CLEC4M | 0.786 | 0.637 | 0.969 | 0.024 |
| CSF3R | 0.642 | 0.432 | 0.953 | 0.028 |
| CXCL2 | 0.530 | 0.389 | 0.722 | 0.000 |
| CXCL5 | 0.096 | 0.015 | 0.607 | 0.013 |
| CXCR4 | 1.270 | 1.024 | 1.574 | 0.029 |
| DEFB128 | 2.496 | 1.191 | 5.231 | 0.015 |
| EGF | 2.191 | 1.071 | 4.482 | 0.032 |
| EGFR | 0.471 | 0.279 | 0.797 | 0.005 |
| EIF2AK2 | 0.600 | 0.402 | 0.897 | 0.013 |
| ENG | 3.778 | 2.050 | 6.960 | 0.000 |
| ESR1 | 0.471 | 0.259 | 0.856 | 0.014 |
| FABP9 | 4.346 | 1.227 | 15.391 | 0.023 |
| FCGR3B | 2.248 | 1.284 | 3.936 | 0.005 |
| FGA | 0.356 | 0.208 | 0.610 | 0.000 |
| FOS | 1.263 | 1.010 | 1.581 | 0.041 |
| GBP2 | 0.442 | 0.240 | 0.811 | 0.008 |
| GCGR | 0.556 | 0.404 | 0.765 | 0.000 |
| GDF10 | 3.511 | 1.481 | 8.326 | 0.004 |
| GDF2 | 0.465 | 0.239 | 0.907 | 0.025 |
| GHR | 0.448 | 0.297 | 0.675 | 0.000 |
| GLP1R | 2.121 | 1.119 | 4.019 | 0.021 |
| GNRH1 | 1.487 | 1.038 | 2.131 | 0.030 |
| HGF | 1.949 | 1.219 | 3.117 | 0.005 |
| HLA-C | 0.637 | 0.412 | 0.985 | 0.043 |
| HNF4A | 0.580 | 0.405 | 0.832 | 0.003 |
| HRG | 0.277 | 0.149 | 0.515 | 0.000 |
| HSP90AA1 | 0.613 | 0.413 | 0.910 | 0.015 |
| IFIH1 | 1.870 | 1.074 | 3.258 | 0.027 |
| IGF2 | 0.642 | 0.440 | 0.939 | 0.022 |
| IL10RA | 1.535 | 1.051 | 2.241 | 0.027 |
| IL11RA | 0.560 | 0.317 | 0.988 | 0.045 |
| IL17C | 1.508 | 1.122 | 2.027 | 0.006 |
| IL17F | 0.203 | 0.059 | 0.692 | 0.011 |
| IL17RA | 0.747 | 0.562 | 0.993 | 0.045 |
| IL18 | 0.607 | 0.384 | 0.960 | 0.033 |
| IL1A | 2.299 | 1.193 | 4.430 | 0.013 |
| IL1RL1 | 2.083 | 1.094 | 3.967 | 0.026 |
| IL21R | 1.750 | 1.003 | 3.054 | 0.049 |
| IL27 | 0.664 | 0.476 | 0.926 | 0.016 |
| IL9 | 4.671 | 1.592 | 13.704 | 0.005 |
| INS-IGF2 | 0.409 | 0.225 | 0.744 | 0.003 |
| IREB2 | 0.639 | 0.423 | 0.964 | 0.033 |
| IRF7 | 1.721 | 1.194 | 2.479 | 0.004 |
| IRF9 | 0.574 | 0.333 | 0.988 | 0.045 |
| JAG1 | 1.615 | 1.120 | 2.329 | 0.010 |
| JAG2 | 1.361 | 1.006 | 1.841 | 0.045 |
| JUND | 0.450 | 0.226 | 0.894 | 0.023 |
| KIR2DS3 | 1.451 | 1.025 | 2.054 | 0.036 |
| KLRC3 | 3.005 | 1.523 | 5.927 | 0.002 |
| KNG1 | 0.570 | 0.331 | 0.981 | 0.042 |
| LCK | 1.608 | 1.029 | 2.511 | 0.037 |
| LEAP2 | 0.487 | 0.331 | 0.718 | 0.000 |
| LECT2 | 0.718 | 0.565 | 0.912 | 0.007 |
| LEFTY1 | 1.647 | 1.067 | 2.540 | 0.024 |
| LEP | 0.621 | 0.402 | 0.957 | 0.031 |
| LILRB3 | 0.544 | 0.380 | 0.778 | 0.001 |
| LTB4R | 1.257 | 1.043 | 1.514 | 0.016 |
| LTBP3 | 2.331 | 1.430 | 3.800 | 0.001 |
| LTBP4 | 2.520 | 1.418 | 4.478 | 0.002 |
| LYN | 1.526 | 1.057 | 2.204 | 0.024 |
| MARCO | 0.387 | 0.195 | 0.768 | 0.007 |
| NCR2 | 4.598 | 1.458 | 14.494 | 0.009 |
| NFKBIB | 0.362 | 0.136 | 0.966 | 0.043 |
| NOD1 | 1.504 | 1.151 | 1.966 | 0.003 |
| NPY | 3.174 | 1.049 | 9.609 | 0.041 |
| NR1H3 | 0.470 | 0.293 | 0.752 | 0.002 |
| NR2F1 | 1.497 | 1.037 | 2.162 | 0.031 |
| NR5A2 | 0.620 | 0.410 | 0.937 | 0.023 |
| NRG3 | 1.695 | 1.133 | 2.537 | 0.010 |
| OBP2A | 5.108 | 2.205 | 11.833 | 0.000 |
| OGFR | 1.590 | 1.085 | 2.330 | 0.017 |
| ORM1 | 0.460 | 0.267 | 0.793 | 0.005 |
| ORM2 | 0.432 | 0.301 | 0.621 | 0.000 |
| OXTR | 0.324 | 0.114 | 0.925 | 0.035 |
| PAK6 | 2.186 | 1.165 | 4.102 | 0.015 |
| PCSK2 | 6.237 | 1.717 | 22.650 | 0.005 |
| PDF | 0.365 | 0.219 | 0.606 | 0.000 |
| PDK1 | 1.883 | 1.021 | 3.472 | 0.043 |
| PGRMC2 | 0.708 | 0.540 | 0.929 | 0.013 |
| PML | 2.718 | 1.481 | 4.987 | 0.001 |
| PMP2 | 5.682 | 2.485 | 12.993 | 0.000 |
| PRLH | 6.791 | 2.287 | 20.162 | 0.001 |
| PROC | 0.464 | 0.295 | 0.730 | 0.001 |
| PSMD6 | 0.442 | 0.238 | 0.819 | 0.009 |
| PSME2 | 0.345 | 0.150 | 0.792 | 0.012 |
| PTGER3 | 1.815 | 1.049 | 3.141 | 0.033 |
| RARA | 0.633 | 0.427 | 0.936 | 0.022 |
| RARB | 1.829 | 1.114 | 3.001 | 0.017 |
| RARG | 1.447 | 1.068 | 1.960 | 0.017 |
| RBP1 | 1.886 | 1.197 | 2.971 | 0.006 |
| RBP5 | 0.545 | 0.307 | 0.966 | 0.038 |
| RELB | 1.463 | 1.062 | 2.017 | 0.020 |
| RLN3 | 3.901 | 1.262 | 12.056 | 0.018 |
| ROBO1 | 1.393 | 1.022 | 1.899 | 0.036 |
| RORA | 0.540 | 0.314 | 0.929 | 0.026 |
| RORC | 0.511 | 0.265 | 0.987 | 0.046 |
| RXRB | 0.322 | 0.127 | 0.815 | 0.017 |
| S100A13 | 2.599 | 1.466 | 4.608 | 0.001 |
| SAA2 | 0.662 | 0.468 | 0.935 | 0.019 |
| SCTR | 1.954 | 1.300 | 2.938 | 0.001 |
| SEMA3B | 1.747 | 1.032 | 2.958 | 0.038 |
| SEMA4B | 1.571 | 1.024 | 2.408 | 0.038 |
| SEMA6D | 1.538 | 1.124 | 2.104 | 0.007 |
| SEMG2 | 0.288 | 0.087 | 0.950 | 0.041 |
| SERPINA3 | 0.543 | 0.370 | 0.797 | 0.002 |
| SHC1 | 4.134 | 1.916 | 8.917 | 0.000 |
| SLC22A17 | 1.744 | 1.167 | 2.606 | 0.007 |
| SLIT2 | 1.472 | 1.086 | 1.994 | 0.013 |
| SLURP1 | 2.182 | 1.093 | 4.355 | 0.027 |
| SPP1 | 1.385 | 1.004 | 1.911 | 0.047 |
| SRC | 1.455 | 1.035 | 2.047 | 0.031 |
| SSTR2 | 0.559 | 0.341 | 0.918 | 0.022 |
| TGFB1 | 1.368 | 1.049 | 1.785 | 0.021 |
| THRB | 0.387 | 0.158 | 0.946 | 0.037 |
| TINAGL1 | 1.596 | 1.024 | 2.486 | 0.039 |
| TMSB4X | 0.611 | 0.388 | 0.963 | 0.034 |
| TNFRSF10A | 2.930 | 1.145 | 7.496 | 0.025 |
| TNFRSF13C | 1.366 | 1.040 | 1.795 | 0.025 |
| TNFRSF14 | 0.606 | 0.390 | 0.941 | 0.026 |
| TNFRSF17 | 0.137 | 0.032 | 0.589 | 0.008 |
| TNFSF14 | 0.562 | 0.331 | 0.955 | 0.033 |
| TNFSF18 | 2.687 | 1.141 | 6.328 | 0.024 |
| TPT1 | 0.547 | 0.340 | 0.881 | 0.013 |
| UMODL1 | 3.909 | 1.235 | 12.375 | 0.020 |
| VTN | 0.369 | 0.202 | 0.674 | 0.001 |
